# Does a single session of theta-burst transcranial magnetic stimulation of inferior temporal cortex affect tinnitus perception?

**DOI:** 10.1186/1471-2202-10-54

**Published:** 2009-05-29

**Authors:** Csaba Poreisz, Walter Paulus, Tobias Moser, Nicolas Lang

**Affiliations:** 1 Department of Clinical Neurophysiology Georg-August University, Göttingen, Germany; 2 Bernstein Center for Computational Neuroscience, Göttingen, Germany; 3 InnerEarLab, Department of Otolaryngology, Georg-August University, Göttingen, Germany; 4 Department of Neurology, Christian-Albrechts University, Kiel, Germany

## Abstract

**Background:**

Cortical excitability changes as well as imbalances in excitatory and inhibitory circuits play a distinct pathophysiological role in chronic tinnitus. Repetitive transcranial magnetic stimulation (rTMS) over the temporoparietal cortex was recently introduced to modulate tinnitus perception. In the current study, the effect of theta-burst stimulation (TBS), a novel rTMS paradigm was investigated in chronic tinnitus. Twenty patients with chronic tinnitus completed the study. Tinnitus severity and loudness were monitored using a tinnitus questionnaire (TQ) and a visual analogue scale (VAS) before each session. Patients received 600 pulses of continuous TBS (cTBS), intermittent TBS (iTBS) and intermediate TBS (imTBS) over left inferior temporal cortex with an intensity of 80% of the individual active or resting motor threshold. Changes in subjective tinnitus perception were measured with a numerical rating scale (NRS).

**Results:**

TBS applied to inferior temporal cortex appeared to be safe. Although half of the patients reported a slight attenuation of tinnitus perception, group analysis resulted in no significant difference when comparing the three specific types of TBS. Converting the NRS into the VAS allowed us to compare the time-course of aftereffects. Only cTBS resulted in a significant short-lasting improvement of the symptoms. In addition there was no significant difference when comparing the responder and non-responder groups regarding their anamnestic and audiological data. The TQ score correlated significantly with the VAS, lower loudness indicating less tinnitus distress.

**Conclusion:**

TBS does not offer a promising outcome for patients with tinnitus in the presented study.

## Background

Tinnitus is an auditory phantom sensation characterised by the perception of elementary sound or noise in the absence of any acoustical stimulation. In chronic tinnitus it is thought that cortical excitability changes, as well as imbalances in excitatory and inhibitory influences, play a distinct pathophysiological role [1]. Increased regional cerebral blood flow (rCBF) in the temporal cortex was measured by positron emission tomography (PET) in patients with tinnitus, compared with healthy controls, on the left side [2] or side contralateral to the tinnitus-affected ear [3]. In line with these results, PET studies using transient reduction of tinnitus by lidocaine also revealed significantly increased rCBF in temporoparietal cortical activity during tinnitus perception [4-6]. However, the laterality of these changes varies between different studies, showing preferentially right hemispheric changes [5,6] or a left hemispheric predominance [7,8]. The imaging studies also indicate increased activity in the secondary auditory cortex, and limbic structures, which may be associated with the emotional distress experienced by tinnitus sufferers [9]. Regarding cortical excitability measures, significantly enhanced intracortical facilitation of the motor cortex [10], was found in tinnitus patients using transcranial magnetic stimulation (TMS). In contrast, a recent study found only a shortening of the duration of the contralateral, and prolongation of the ipsilateral cortical silent period in tinnitus patients [11].

Modulation of cortical excitability by means of repetitive transcranial magnetic stimulation (rTMS) has developed into a major research focus in neurophysiology and as a potential treatment in neuropsychiatry [12]. Generally, high frequencies of rTMS facilitate [13], whereas low frequencies inhibit cortical excitability [14]. rTMS over the auditory cortex was recently introduced to modulate tinnitus perception (for review see [15,16]). Single sessions of rTMS were applied at high frequencies and resulted in a short-lasting but significant improvement [17-19], whereas low frequencies have been used for approximately 5- or 10-day treatment trials and showed a long-lasting reduction in symptoms [20-26]. Comparison of the effect of high- and low-frequency rTMS showed that brief high frequency rTMS (3 s of 10 Hz) has no effect, whereas prolonged low frequency rTMS (1200 s 1 Hz) has a significant effect on tinnitus [27]. In a study by De Ridder et al. [28], 114 chronic tinnitus sufferers (the highest number of any study so far) showed surprisingly, that both the high and low-frequency rTMS applications were effective. Here the efficacy of various rTMS frequencies was significantly correlated with the duration of the tinnitus; rTMS at higher frequencies is more effective for short duration tinnitus and low frequencies for long standing tinnitus. In addition the amount of improvement correlated negatively with tinnitus duration [28], which was confirmed by Plewnia et al. [25]. The largest double-blind parallel study compared the effects of different frequencies of rTMS (1 Hz, 10 Hz, 25 Hz and sham (occipital, 1 Hz)), given daily over the left temporoparietal cortex for 2 weeks [29]. There was no significant difference between the responses to different frequencies of rTMS, but were significantly effective when compared to sham. Preconditioning the temporal cortex with high-frequency rTMS before low-frequency stimulation did not result in more pronounced effects [30]. Combination of high-frequency prefrontal and low-frequency temporal rTMS compared to low-frequency treatment showed that directly after therapy there was an improvement of the TQ-score for both groups, but no differences between groups. An evaluation after 3 months revealed remarkable beneficial effects from the use of combined prefrontal and temporal rTMS treatment [31].

Recently a specific rTMS paradigm, namely theta-burst stimulation (TBS) was developed [32] to modulate human primary motor cortex (M1) excitability. The authors distinguished three stimulation patterns with differential effects on M1 excitability. Continuous TBS (cTBS) was found to inhibit, whereas intermittent TBS (iTBS) facilitated M1 excitability. Interestingly, intermediate TBS (imTBS) had no effect on MEP amplitudes. Besides M1, TBS has also been shown to influence the excitability of the human premotor [33], visual [34] and primary somatosensory cortices [35].

Recently, it has been demonstrated that rTMS applied in bursts of five pulses at 50 Hz repeated at 5 Hz over the auditory cortex has significantly stronger effects on narrow band/white noise tinnitus than tonic 5 Hz stimulation [36]. Furthermore, by using burst stimulations in different frequencies, it has been shown that burst neuromodulation is more powerful than tonic neuromodulation [37].

The aim of the current study was to investigate the effects of all three TBS paradigms in a randomized, single-blinded cross-over design on tinnitus perception in patients with chronic tinnitus. On the basis of previous reports regarding the use of conventional low- and high-frequency rTMS in tinnitus [17,19,28] we hypothesized that single sessions of 40–190 sec TBS would also be able to produce a transient attenuation of tinnitus perception. This hypothesis was supported by a recent report that TBS results in comparable after-effects on M1 excitability when compared with conventional high- and low-frequency rTMS [38], yet being still more applicable for blinded studies and having a protocol of much shorter duration.

## Results

**Figure 1:**
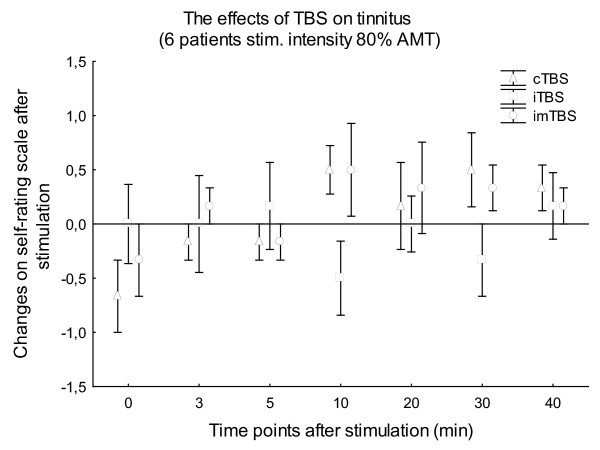
**Figure 1 shows the means of the changes on the self-rating scale in the first 6 patients**. Stimulation intensity was 80% AMT, effects were observed during the first 40 min after TBS. Vertical bars denote SEM.

**Figure 2:**
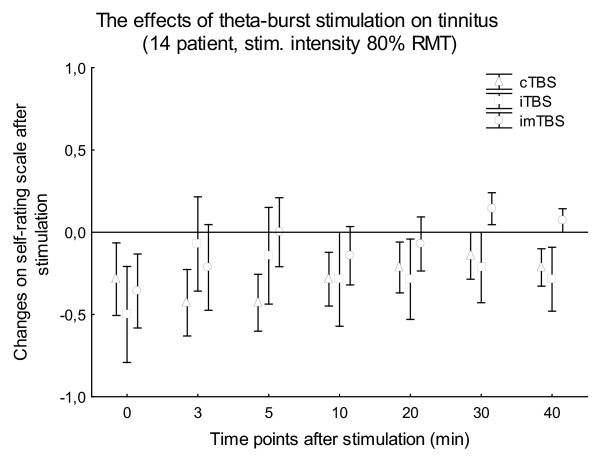
**Figure 2 shows the means of the changes on the self-rating scale in 14 patients**. Stimulation intensity was 80% RMT, effects were observed during the first 40 min after TBS. Vertical bars denote SEM.

**Figure 3:**
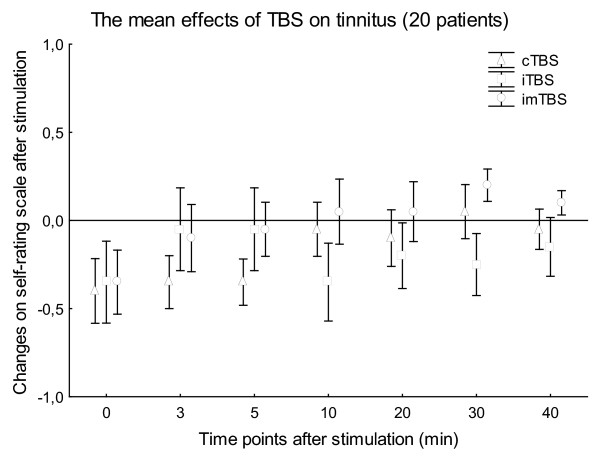
**Figure 3 shows the means of the changes on the self-rating scale in all of the 20 patients**. Stimulation intensity was varied between 80% AMT (6 patients) and 80% RMT (14 patients), effects were observed during the first 40 min after TBS. Vertical bars denote SEM.

The mean stimulation intensities were 37.56 ± 4.30% (80% AMT) and 41.93 ± 5.34% (80% RMT) of the maximal stimulator output, respectively. This resulted in mild to moderate twitching of facial muscles on the left side of the head, observed in all patients during the stimulation. None of the patients had any type of epileptic symptoms during or after TBS. Wilcoxon matched pairs tests revealed no significant difference in the baseline VAS values between the 3 stimulation types in the case of all patient or patients stimulated with 80% AMT or 80% RMT (p > 0.05). The non-parametric Friedman ANOVAs resulted in no significance for STIMULATION in the case of TBS with 80% AMT (6 patients; p > 0.23) (Fig. 1) and 80% RMT (14 patients; p > 0.11) (Fig. 2) at any time point after stimulation. The non-parametric Friedman ANOVAs, calculated for all the 20 patients for every time point separately, also showed no significant effect of STIMULATION (p > 0.15) (Fig. 3).

**Figure 4:**
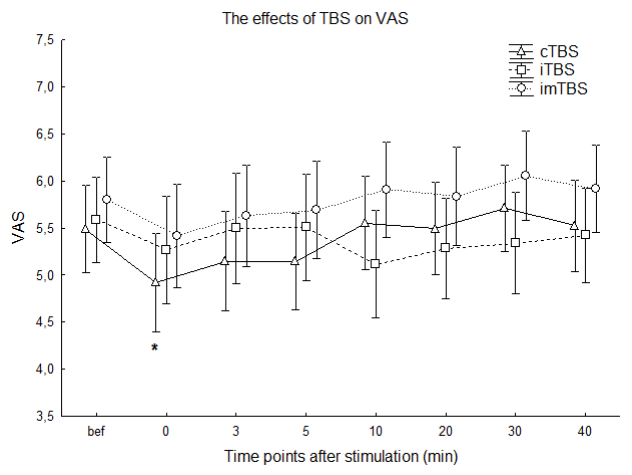
**Figure 4 shows the means of the VAS at every time point before and after stimulation**. Please note, that the after values are calculated by converting the changes of the NRS into the VAS using the formula VAS_t _= VAS_bef_+VAS_bef_/4*NRS_t _in case of improvement (negative values of NRS) or VAS_t _= VAS_bef_+(10-VAS_bef_)/3*NRS_t _in case of worsening of the symptom, for each time point (_t_) separately. * indicate significant difference compared to baseline Wilcoxon matched pairs test (significance level p < 0.05); Vertical bars denote SEM.

There were no significant differences when we compared the calculated VAS scores between the two groups with different stimulation intensities (80% AMT n = 6; and 80% RMT n = 14) at any time point for all TBS protocols (p > 0.1, Mann-Whitney U test). Therefore further analyses were done involving all the 20 patients. Wilcoxon matched pairs tests calculated for each TBS protocol separately, resulted in a significant difference only in case of cTBS between baseline and the time point immediately after the stimulation (n = 20; p = 0.015) Fig 4.

**Figure 5:**
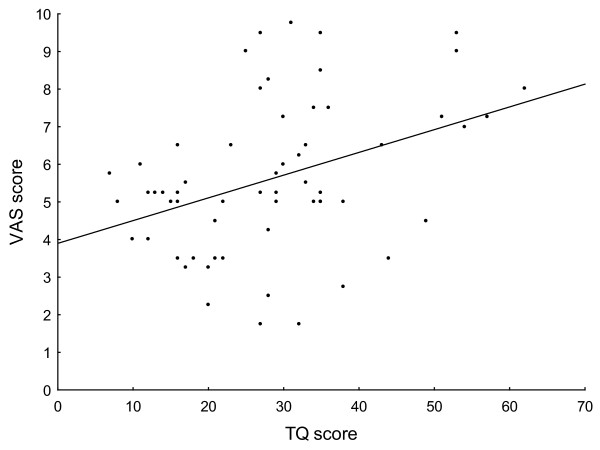
**Figure 5 shows the correlation between the tinnitus severity (TQ score) and the score from the VAS**. TQ and VAS scores were measured prior to each TBS sessions (3-3 scores from all 20 patients). The significant correlation (r = 0.39, p = 0.02) suggests that a lower VAS score indicates lower tinnitus related distress, and therefore may indicate that both methods are good for monitoring the actual state of the patients.

We did not find any significant difference in tinnitus duration (p = 0.47), dominant site of the symptom (p = 0.63), hearing loss (p = 0.42), TQ score (p = 0.13) or stimulation intensity (p = 0.24) between the responder (n = 11) and non-responder groups (n = 9). The tinnitus distress (TQ score) correlated significantly with the VAS (Spearman R = 0.349, p = 0.006) suggesting that TQ score is related to the actual tinnitus perception (Fig. 5).

## Discussion

In the present study we could not find any significantly different effect on tinnitus perception for the different types of TBS applied to the inferior temporal cortex, either at the lower intensities of 80% AMT, nor at the higher intensities of 80% RMT. On an observational level, a slight to marked reduction of the symptoms was seen in about half of the patients, irrespective of the type of TBS, which was only significant immediately after cTBS compared to baseline. We did not find any significant difference between the responder and non-responder groups regarding the duration or laterality of the symptoms, or the amount of hearing loss. The intensity of the stimulation also did not significantly differ between the two groups that may indicate that the observed slight effects are not intensity dependent, and that the loudness of the noise evoked by the stimulation did not influence the patients. The correlation between the TQ and VAS scores (at lower TQ lower VAS score) suggests that both methods may be good for monitoring the actual state of the patients. In addition, we found a relatively high rate of unpleasant side effects of TBS which was the result of the additional stimulation of peripheral nerves and orofacial muscles. Regarding safety concerns i.e. seizures; TBS with 80% RMT over T3 was not problematic.

Why did single sessions of TBS have no significant effect on tinnitus? The first possible explanation is that TBS had no effect in our study over the temporal cortex because it could not reach the tinnitus-related areas or was not sufficient to induce excitability changes in these areas. We chose to stimulate all our patients on the left side of the head, over the T3 EEG-electrode position, irrespective of their tinnitus- affected side, as the primary studies reported positive effects on tinnitus after rTMS over T3 [17,19] or very close to it [18]. Moreover, Kleinjung et al [23] found that in their tinnitus patients there was a left hemispheric predominance in rCBF examined by PET. However, later studies chose to stimulate the temporoparietal cortex (halfway between T3 and P3 [29] or halfway between C3 and T3 [18]) or used the individual fMRI or PET data for neuronavigation, and showed more consistent after-effects. It may well be that the stimulation site we used was not adequate to attenuate tinnitus perception, which would explain our negative results.

Regarding the intensity of the TBS, it was first set to 80% of the AMT [32], however after we amended the original Huang protocol to 80% of the RMT, which resulted in a stronger but still subthreshold stimulation. However, even this enhanced stimulation intensity did not result in better effects on tinnitus perception (Fig. 1. and Fig. 2.). Stimulation of the temporal cortex with TBS at RMT or above, or using a higher number of impulses was regarded as unsafe by our own safety guidelines, and due to the need for clear safety limits for TBS, safety limits of conventional rTMS [39] should also be applied.

If TBS applied over the left inferior temporal cortex was actually not effective on tinnitus, we should consider that all of our non-significant but not negligible observed effects were caused by the placebo effect. It is important to mention that the placebo effect is high in most of the clinical rTMS studies, regardless of the paradigm used [12]. We were not able to use a proper sham condition, as the currently available methods (for example using a sham coil or tilting the coil at 90° angle on the wing [40]) results in no additional stimulation of the peripheral nerves and orofacial muscles. Therefore we decided to use imTBS as an „active" sham condition, although it must be questioned whether this protocol can really be considered ineffective. Still, with the exception of Huang and colleagues, who published the first series of TBS experiments on the motor cortex [32] and stated that imTBS has no effect, there has been no other study, which has confirmed this. In a recent study we found, that imTBS applied over the primary somatosensory cortex has a significant effect on the N2 component of the laser-evoked potential [35], but not the sham protocol. These results indicate that imTBS may be an active condition at least when applied to non-motor cortical areas. Therefore, another possible explanation as to why TBS had no significant effect on tinnitus in our study may be that there was no adequate placebo condition; which is another limitation of our study. Furthermore, our study design did not allow the direct comparison of all of the influences on tinnitus perception with baseline values. Therefore we converted the NRS scales to the VAS, which allows a comparison between the observed changes to the baseline. Here we found a significant improvement, but only immediately after cTBS (Fig 4). Since we did not use a conventional sham stimulation it is impossible to decide whether it is a specific or unspecific effect of cTBS.

The neuronal mechanisms of theta burst paradigm are still highly speculative. The results of the experiments using single trains of TBS suggest that in the human motor cortex TBS produces a mixture of facilitatory and inhibitory effects on synaptic transmission [41]. Huang and Rothwell proposed, that facilitation develops faster than inhibition; thus in case of inhibitory cTBS, several seconds after an initial facilitation, the inhibition overrides this effect whereas iTBS uses only the early excitatory effect in the initial 2 s. Most likely, the underlying mechanisms will involve many of the basic elementary mechanisms described previously in the LTP/LTD literature [42]. In line with these results, recently TMS was applied to the cat visual cortex and the neural and hemodynamic consequences were evaluated [43]. Short TMS pulse trains elicited initial activation (~1 minute) and prolonged suppression (5 to 10 minutes) of neural responses demonstrating long-lasting neural responses to TMS. Furthermore, TMS disrupted the temporal structure of activity by altering phase relationships between neural signals.

It is possible that the difference in effectiveness observed between TBS protocols on motor and sensory cortices could be due to differences in the physiological and functional states of the stimulated cortex. In a recent animal study the expression of the two immediate early gene (IEG) proteins, which are involved in both LTP and LTD like mechanisms, namely c-Fos and zif268 were examined in the rat brain after 1 Hz, 10 Hz rTMS and iTBS [44]. The cortical expression of both IEGs was specifically changed in an rTMS-dependent manner. In addition they found that cortical induction of c-Fos and zif268 expression by rTMS differed depending on the type of cortical area, indicating that neuronal networks intrinsic to certain areas or those being involved in connecting cortical and subcortical areas might have been differentially entrained by the temporal structure of the magnetoelectric stimulation.

Concerning measuring cortical excitability as a way of monitoring of the functional status, in tinnitus, clinical improvement was associated with an increase in intracortical inhibition, intracortical facilitation and a prolongation of the cortical silent period [45]. However, when patients were compared with healthy controls, only minor changes were found [10,11]; such minor changes seem unlikely to be an important factor in the development of clinical symptoms. Furthermore, several studies have shown that both low- and high-frequency rTMS reduce tinnitus [17,28,29,37] indicating that TMS effects on motor cortex excitability are different from TMS effects on tinnitus perception. One session of rTMS has only very short-lasting effects on tinnitus perception [28]. Burst stimulations have been found to be more powerful than tonic rTMS [37], and it was suggested that burst stimulation modulates the extralemniscal and lemniscal systems, whereas tonic stimulation modulates only the lemniscal system [36]. However, these burst stimulations vary within many parameters (not only the frequency) from the TBS data published by Huang et al. [32] and that could also be a possible reason as to why our results differ from previous findings. Furthermore women experience greater suppression of their tinnitus with burst stimulation than men [37] and since we had only two women, it could influence our results. Our study design and results do not allow us to draw conclusiosn about the neuronal mechanisms of TMS on the temporal cortex, but may show that the effects of TMS on tinnitus are not directly mediated by TMS induced modulation of excitability in the stimulated cortical area.

## Conclusion

In our study, only cTBS resulted in significant immediate aftereffects on tinnitus perception. Still, a slight to marked improvement was observed by about the half of our patients, but irrespective of the type of TBS. The absence of any other significant effects in this study could depend on the lack of a reliable sham stimulation, or caused by the inadequate stimulation site. It is important to note that in previous studies using high-frequency suprathreshold rTMS, the improvement in tinnitus was observed by about 42–68% of the stimulated patients. According to the recent results of rTMS applied in alpha-, beta-, and theta-bursts [37], new types of burst stimulation protocols may be more effective in tinnitus. Regarding TBS, neuronavigated stimulation, more stimulation sessions with longer poststimulation observation period, and the development of reliable sham stimulation may be necessary to evaluate the impact of this technique on tinnitus. Future studies should be estimating the effects of TMS on neural activity from the amplitude of cortical evoked potentials.

## Methods

### Subjects

Thirty-three patients with chronic tinnitus participated, and twenty patients (18 male, mean age 47.6 ± 12.68 years) completed the study. Thirteen patients failed to complete the study for varying reasons. One male patient complained of persistent headache and one female patient felt that the stimulation was too unpleasant and did not want to continue the study after sessions of TBS with an intensity of 80% AMT. Regarding the enhanced TBS intensity (80% RMT) three female patients experienced worsening of tinnitus after the first stimulation, four patients found the stimulation too unpleasant, one experienced headache after the first stimulation and three patients left the study without citing any specific reason.

All of the participants were informed about all aspects of the experiments and signed an informed consent. The study protocol conformed to the Declaration of Helsinki and was approved by the Ethics Committee of the University of Göttingen.

The duration of the tinnitus was 94.35 ± 78.66 months (mean ± SD), and ranged between 8–276 months. Symptoms were bilateral or located in the head in 12 patients, the remainder had predominantly left (4 patients) or right-sided (4 patients) tinnitus. The tinnitus severity was 31.7 ± 12.97 ranging from 10 to 55 at the beginning of the study according to the Tinnitus-Questionnaire (TQ) developed by Goebel and Hiller [46].

At the beginning of the study, ear microscopy, pure-tone audiometry, tympanometry, stapedius reflex testing and auditory brainstem responses were examined in all of the patients. Pure-tone audiometry showed normal hearing in 3 individuals (less than 10 dB SPL at all frequencies); the remainder had mild (40 dB, 2 patients), moderate (40–65 dB, 9 patients) or moderately severe (70 dB, 5 patients) symmetrical or asymmetrical sensorineural hearing loss (given at the worst frequency SPL). One patient was deaf in one ear (for details see additional file 1: Tabl1.xls).

Patients taking anticonvulsant or tranquilizer medication, and patients with neurological, psychiatric or severe systemic disease or with a history of surgery or injury of the ears, were excluded, as well as patients with any contraindications for TMS.

### Theta-burst stimulation (TBS)

TBS was applied over the left inferior temporal cortex with a standard figure-of-eight-coil (MCF-B65) and MagPro stimulator (Medtronic, Denmark) with an anterior-posterior-anterior current flow in the coil. The centre of the coil was positioned over the T3 EEG-electrode position (10–20 international EEG system) with the handle of the coil pointing backwards. In our pilot study we stimulated 6 patients with an intensity of 80% of active motor threshold (AMT), in the remaining 14 patients the stimulus intensity was set to 80% of resting motor threshold (RMT).

For RMT and AMT determination, the coil was placed tangentially onto the scalp over the optimal representation of the right abductor digiti minimi muscle (ADM), with the handle pointing backwards and laterally in a 45° angle. MEPs of the right ADM were recorded by surface Ag-AgCl electrodes in a belly-tendon-montage. The signals were amplified and filtered (2 Hz-3 kHz, sampling rate of 5 kHz), digitalized with a micro-1401 AD converter (Cambridge Electronic Design, Cambridge, UK) and recorded by a computer using the SIGNAL software (Cambridge Electronic Design, version 2.13). RMT was defined as the lowest stimulus intensity, which elicited a peak-to-peak MEP-amplitude of app. 50 *μ*V or more in the resting muscle in at least 3 out of 6 recordings. Complete muscle relaxation was controlled though auditory and visual feedback of EMG activity. AMT was defined as the minimum intensity at which at least 3 out of 6 consecutive stimuli elicited a MEP of app. 200 μV in amplitude during moderate contraction of the ADM. Thresholds were measured before each stimulation conditions.

The pattern of TBS consisted of bursts containing 3 pulses at 50 Hz which (triads) were repeated at 200 ms intervals (i.e. 5 Hz) for up to 600 pulses for 40 s continuously (cTBS), or triads repeated at 200 ms intervals for 2 s intermittently in every 10 s up to 600 pulses (iTBS). In the case of imTBS the triads were repeated at 200 ms intervals for 5 s, in every 15 seconds up to 600 pulses [32]. The TBS protocols were configured and triggered by a computer using the SIGNAL software (Cambridge Electronic Design, version 2.13). The order of the three TBS protocols was pseudorandomised and balanced between subjects and they were blinded for TBS conditions. Stimulation sessions were separated from each other by at least 5 days. Subjects wore ear plugs only during the stimulation to reduce auditory artifacts accompanying TMS.

### Psychophysical evaluation and experimental design

At the beginning of each stimulation session patients were asked to fill in the Tinnitus Questionnaire (TQ,) [46] and indicate the actual loudness of their tinnitus sensation as per the visual analog scale (VAS). VAS was a line (20 cm in total) where the start point on the left side meant no tinnitus and the right end point indicated the strongest conceivable tinnitus. During the data analysis VAS scores were converted to a scale from 0 to 10 where each 0.5 cm of the 20 cm line meant 0.25 in the VAS rating. The VAS was used to monitor the actual distress and tinnitus loudness before all sessions. After this the motor threshold was determined and TBS was performed.

After each train, the relative *change *of tinnitus was evaluated with a numerical self-rating scale (NRS) (-4 to 3) with 0 corresponding to no, -1 to slight, -2 to marked, -3 to strong reduction, and -4 to complete suppression, whereas +1 to slight, +2 to marked and +3 to strong worsening of tinnitus. The scores of the self-rating scale were assessed immediately after stimulation and at 3, 5, 10, 20, 30 and 40 minutes after stimulation by all patients.

### Statistical analysis

Statistical analysis was performed by Statistica computer software [StatSoft, Inc. (2006). STATISTICA (data analysis software system), version 7.1. ]. Non-parametric Friedman-ANOVAs were calculated using the values of the numerical self-rating scale values (-4 to 3) to compare the different stimulation effects (cTBS, iTBS and imTBS) at every time point after the stimulation separately. We calculated Friedman-ANOVAs separately for the two TBS intensities (80% AMT and 80% RMT) and an all in ANOVA irrespective of the stimulation intensity.

Since the NRS values (-4 to 3) could not permit a comparison between the time course of the effects in the absence of any baseline; we could not find any difference between the aftereffects of the different types of TBS. It was therefore necessary to convert the two scales. We converted the NRS values (-4 to 3) to the VAS using the formula VAS_t _= VAS_bef_+VAS_bef_/4*NRS_t _in case of improvement (negative values of NRS) or VAS_t _= VAS_bef_+(10-VAS_bef_)/3*NRS_t _in case of worsening of the symptoms, for each time point (_t_) separately. The effects of the stimulus intensity (80% AMT or 80% RMT) were analyzed first using the Mann-Whitney U test (significance level p < 0.05) between the two groups. To evaluate the statistical effects of each TBS protocols in time Wilcoxon matched pairs test (significance level p < 0.05) were used to compare the baseline VAS values with the calculated VAS values for each time points separately.

We divided our patients according to their responses to cTBS and iTBS into two groups (see additional file 1: Tabl1.xls). Comparing the audiological and anamnestic data, like tinnitus duration, the dominant site of tinnitus (in the case of bilateral tinnitus, the site which was more affected), hearing loss (on the side of the tinnitus), tinnitus distress (scores from the TQ), intensity of the TBS (given in % of the maximal stimulator output) between the responder and non-responder groups were done using a Mann-Whitney U test (significance level p < 0.05). Furthermore, we rated the tinnitus distress (TQ scores) to the VAS scores which were evaluated three times by all patients before the stimulation sessions, and were therefore independent from the type of TBS and were correlated in one analysis. For correlation analyses the non-parametric Spearman Rank Order correlation (p < 0.05) was used.

## Authors' contributions

CP recruited the patients and performed audiologic measurements, carried out the stimulations and drafted the manuscript; WP participated in the conception and the design of the study and helped draft the manuscript; TM participated in the design of the study, helped to recruit the patients and to perform audiologic measurements; NL postulated the study, participated in its design and coordination and helped to draft the manuscript. All authors read and approved the final manuscript.

## Supplementary Material

Additional file 1 **Table 1**. The main characteristic of patients and the individual stimulation results. Click here for file
